# Testing a MultiTEP-based combination vaccine to reduce Aβ and tau pathology in Tau22/5xFAD bigenic mice

**DOI:** 10.1186/s13195-019-0556-2

**Published:** 2019-12-17

**Authors:** Hayk Davtyan, Armine Hovakimyan, Sepideh Kiani Shabestari, Tatevik Antonyan, Morgan A. Coburn, Karen Zagorski, Gor Chailyan, Irina Petrushina, Olga Svystun, Emma Danhash, Nikolai Petrovsky, David H. Cribbs, Michael G. Agadjanyan, Mathew Blurton-Jones, Anahit Ghochikyan

**Affiliations:** 1 Department of Molecular Immunology, Institute for Molecular Medicine, Huntington Beach, CA USA; 2 Institute for Memory Impairments and Neurological Disorders, University of California, Irvine, Irvine, CA USA; 3 Sue and Bill Gross Stem Cell Research Center, University of California, Irvine, Irvine, CA USA; 4 Department of Neurobiology and Behavior, University of California, Irvine, Irvine, CA USA; 5 Current address: Department of Pharmaceutical Sciences, College of Pharmacy, University of Nebraska Medical Center, Omaha, NE USA; 6 School of Biological Sciences, University of California, Irvine, Irvine, CA USA; 7 Flinders University and Vaxine Pty Ltd, Adelaide, Australia

**Keywords:** MultiTEP platform, Alzheimer’s disease, Protein epitope vaccine, Antibody, Adjuvant, Bigenic mice, T5x mice, Aβ_42_ and tau pathology

## Abstract

**Background:**

Alzheimer disease (AD) is characterized by the accumulation of beta-amyloid (Aβ) plaques and neurofibrillary tangles composed of hyperphosphorylated tau, which together lead to neurodegeneration and cognitive decline. Current therapeutic approaches have primarily aimed to reduce pathological aggregates of either Aβ or tau, yet phase 3 clinical trials of these approaches have thus far failed to delay disease progression in humans. Strong preclinical evidence indicates that these two abnormally aggregated proteins interact synergistically to drive downstream neurodegeneration. Therefore, combinatorial therapies that concurrently target both Aβ and tau might be needed for effective disease modification.

**Methods:**

A combinatorial vaccination approach was designed to concurrently target both Aβ and tau pathologies. Tau22/5xFAD (T5x) bigenic mice that develop both pathological Aβ and tau aggregates were injected intramuscularly with a mixture of two MultiTEP epitope vaccines: AV-1959R and AV-1980R, targeting Aβ and tau, respectively, and formulated in Advax^CpG^, a potent polysaccharide adjuvant. Antibody responses of vaccinated animals were measured by ELISA, and neuropathological changes were determined in brain homogenates of vaccinated and control mice using ELISA and Meso Scale Discovery (MSD) multiplex assays.

**Results:**

T5x mice immunized with a mixture of Aβ- and tau-targeting vaccines generated high Aβ- and tau-specific antibody titers that recognized senile plaques and neurofibrillary tangles/neuropil threads in human AD brain sections. Production of these antibodies in turn led to significant reductions in the levels of soluble and insoluble total tau, and hyperphosphorylated tau as well as insoluble Aβ_42_, within the brains of bigenic T5x mice.

**Conclusions:**

AV-1959R and AV-1980R formulated with Advax^CpG^ adjuvant are immunogenic and therapeutically potent vaccines that in combination can effectively reduce both of the hallmark pathologies of AD in bigenic mice. Taken together, these findings warrant further development of this vaccine technology for ultimate testing in human AD.

## Background

Alzheimer’s disease (AD) is a complex and multifactorial disease involving genetic and environmental risk factors that together lead to the progressive accumulation of two hallmark pathologies: β-amyloid plaques and neurofibrillary tangles (NFTs). Although many clinical trials have aimed to reduce β-amyloid and, more recently, to target the accumulation of tau that drives NFT formation, debate remains regarding which of these pathologies represents the most tractable target, and the precise timing for these potential treatments. Recent longitudinal analyses of participants within the Alzheimer’s Disease Neuroimaging Initiative (ADNI) demonstrated evidence of synergism between Aβ and phosphorylated tau (p-tau) suggesting these pathologies may interact to trigger the progression from amnestic mild cognitive impairment (MCI) subjects to AD dementia [1, 2]. PET imaging studies suggest that Aβ deposits start decades before dementia onset, and may or may not precede tau pathology, with the latter correlating better with symptom onset and the degree of dementia [3, 4]. According to the modified amyloid cascade model proposed by Jack et al. [5–7], primary age-related tauopathy (PART) develops universally as a function of aging and, by itself, produces no or only mild cognitive symptoms. Aβ deposition occurs independently in the neocortex and induces or facilitates the spread of pathological tau, perhaps by promoting the production of pathological tau strains [8]. Pathological tau is directly associated with neurodegeneration, which in turn drives cognitive decline. In this model of AD, Aβ does not directly cause cognitive symptoms but is still central to disease pathogenesis as a dominant driver of downstream pathological processes including tau pathology [6, 7]. Indeed, a recent study utilizing serial PET measurements of both amyloid and tau provides strong additional support for this hypothesis [9].

This synergistic model suggests that combinatorial/multi-target therapies directed at the accumulation of both amyloid and tau pathologies may be more effective in the treatment of AD than previously tested unimodal approaches.

Recently, we demonstrated that the combination of AV-1959R and AV-1980R vaccines targeting Aβ and tau, respectively, induced robust antibody responses against various forms of both Aβ and tau pathological molecules in wildtype mice [10]. Both of these vaccines are based on the MultiTEP platform that consists of a string of 12 non-self, pathogen-derived T helper (Th) epitopes to which Aβ B cell epitopes (AV-1959R) and tau B cell epitopes (AV-1980R) are attached. A dual vaccine expressing both Aβ and tau B cell epitopes (AV-1953R) generated similar concentrations of anti-Aβ antibodies, but significantly lower concentrations of anti-tau antibodies compared to mice vaccinated with a combination of AV-1959R and AV-1980R [10], suggesting a mixed vaccine approach may be preferred.

Here, we tested the therapeutic efficacy of co-formulated vaccines targeting Aβ and tau administered simultaneously in combination with Advax^CpG^ adjuvant in the Tau22/5xFAD (T5x) mouse model of AD. T5x bigenic mice [11] were generated by crossing two existing and well-characterized transgenic models, 5xFAD [12] and THY-Tau22 [13], and were previously shown to develop highly aggressive Aβ and tau pathology, and thus likely represent a useful model for testing potential AD therapies. The bigenic T5x mice exhibit an approximately threefold increase in misfolded and hyperphosphorylated tau over the parental Tau22 strain, further supporting the hypothesis that Aβ accelerates tau pathology [11].

## Materials and methods

### Mice

Thy-Tau22-5xFAD (T5x) double transgenic AD mice were generated as described in [11]. Briefly, Thy-Tau22 mice express human 4 repeat tau with two frontotemporal dementia-associated point mutations (G272V and P301S) under control of the neuronal driven promoter Thy1.2 and are maintained on a C57Bl6/J background [13]. The 5xFAD mice used in this study are also maintained on a congenic C57Bl6/J and co-expresses human amyloid precursor protein (APP695) carrying the Swedish, Florida, and London mutations and a human presenilin-1 (PS1) transgene carrying the M146L and L286V mutations under the Thy-1 promoter. Both APP and PS1 transgenes are co-integrated and thus co-inherited. Heterozygous Thy-Tau22 and 5xFAD mice were crossed to create Thy-Tau22-5xFAD (T5x) mice that were genotyped via PCR amplification of human tau, PS1, and APP transgenes. Both female and male animals were used in this study and sex-dependent effects examined. All animals were housed in a temperature and light cycle-controlled facility, and their care was under the guidelines of the National Institutes of Health and an approved IACUC protocol at University of California, Irvine.

### Epitope vaccines and purification of proteins

To prepare two recombinant proteins, minigenes encoding 3Aβ_1–11_-MultiTEP or 3Tau_2–18_-MultiTEP were cloned into the modified *Escherichia coli* expression vector pET11 (for AV-1959R; Novagen, MA) or pET24a (for AV-1980R; Novagen, MA) in frame with 6xHis-Tag at the C-terminus. Gene encoding 2N4R tau protein was amplified from human whole brain Marathon®-Ready cDNA library (Clontech) using primers 5′-catatggctgagccccgccaggagttcgaagtgatg (forward) and 5′-ctcgagtcacaaaccctgcttggccagggaggcagac (reverse) and cloned into the pET24a + *E. coli* expression vector in frame with 6xHis-tag at the C-terminus using restriction sites NdeI and XhoI. DNA sequencing was performed to confirm that the generated plasmids contained the correct sequences. Recombinant proteins were purified from *E. coli* BL21 (DE3) cells transformed with pET11/3Aβ_1–11_-MultiTEP, pET24a/3Tau_2–18_-MultiTEP, or pET24a/Tau plasmids as described [10, 14, 15] for epitope vaccines and in [16] for Tau protein. The final recombinant protein was analyzed in 10% Bis-Tris gel electrophoresis (NuPAGE Novex Gel, Invitrogen, CA). Protein bands were visualized by Coomassie dye, and specificity of the bands was confirmed by Western blot (WB) using 6E10 and anti-tau_2–18_ 1C9 monoclonal antibodies [10]. The level of endotoxin was measured using E-TOXATE kits, as recommended by the manufacturer (Sigma, St Louis, MO).

### Preparation of oligomeric recombinant tau

Oligomeric forms of tau protein were prepared as described by Combs et al. [17]. Briefly, arachidonic acid in ethanol was added to recombinant tau protein in polymerization buffer (5 mM DTT, 100 mM NaCl, 100 μM EDTA, and 10 mM HEPES at pH 7.64) to a final concentration of 75 μM in order to induce tau polymerization. The reaction was allowed to proceed overnight, and the extent of aggregation was confirmed by western blotting. The aggregated tau sample was aliquoted and was stored at − 80 °C until used in SPR assay.

### Experimental protocols

**Fig. 1:**
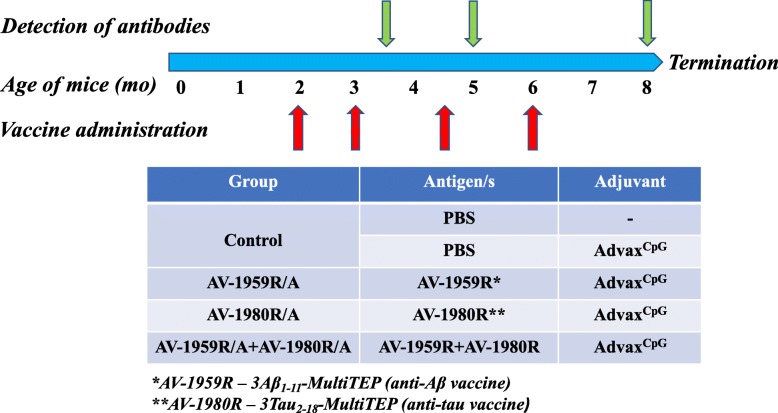
Design of immunization study of T5x mice. Experimental protocol in T5x mice vaccinated with AV-1959R and AV-1980R proteins separately or mixed together formulated in Advax^CpG^ adjuvant. Control mice were injected either with PBS or Advax^CpG^ adjuvant only

Three groups of T5x mice were immunized with AV-1959R (20 μg/per mouse/per injection), AV-1980R (20 μg/per mouse/per injection), or a mixture of AV-1959R and AV-1980R proteins (20 μg protein/mouse/injection), all formulated with Advax^CpG^ adjuvant (Vaxine Pty Ltd., Adelaide Australia) at 1 mg/mouse/injection. Control groups of T5x mice were injected with Advax^CpG^ adjuvant only or PBS. All mice were injected four times intramuscularly. Sera were collected 14 days after third immunizations, and anti-Aβ and anti-tau antibody responses were analyzed. At age of 8 months old, mice were terminated and brains were collected for biochemical and immunohistological analysis. More detailed experimental protocols are provided in Fig. 1.

### Detection of Aβ- and tau-specific antibodies

The concentrations of anti-Aβ and anti-tau antibodies in serum were determined by ELISA, as described previously [15, 18, 19]. To measure anti-Aβ and anti-tau antibody concentration, plates were coated with 1 μg/per well Aβ_42_ peptide (American Peptide, CA), tau_2–18_ peptide (GenScript, NJ), or full-length recombinant tau protein (purified at The Institute for Molecular Medicine, Huntington Beach, CA), respectively. Anti-Aβ and anti-tau antibody concentrations were calculated using a calibration curve generated with affinity-purified polyclonal antibodies from sera of mice vaccinated with AV-1959R/A and AV-1980R/A, respectively. HRP-conjugated anti-mouse IgG (Jackson ImmunoResearch Laboratories, ME) was used as secondary antibody.

### Epitope mapping of tau-specific antibodies

Epitope mapping of anti-tau antibodies was performed by “alanine scanning” using competitive ELISA. Briefly, 17 peptides spanning tau_2–18_ sequence, but possessing one alanine substitution in each position were synthesized. Ninety-six-well plates (Immulux HB; Dynex Technologies, Inc., VA) were coated with 1 μg/well (in 100 μl; Carbonate-Bicarbonate buffer, pH 9.6, o/n at 4 °C) tau_2–18_ peptide (GenScript, NJ). Next day coated plates were blocked with blocking buffer (3% dry, non-fat milk in TBST, 300 μl/well). Serial dilutions of reference wild type (tau_2–18_) or mutated test peptides (corresponding to 0.02 μM, 0.1 μM, 0.5 μM, 2.5 μM, 5 μM, 12.5 μM, and 25 μM final concentrations) were incubated with immune sera diluted 1: 300,000 (corresponding to the linear region of the curve for binding to Tau_2–18_ peptide) for 1.5 h at 37 °C. After incubation, 100 μl of antibody/peptide mixture was added into the wells. HRP-conjugated goat anti-mouse IgG (1:2500; Jackson ImmunoResearch Laboratories, PA) were used as secondary antibodies. The reaction was developed by adding *3,3′,5,5′tetramethylbenzidine* (TMB) (Pierce, IL) substrate solution and stopped with 2 M H_2_SO_4_. The optical density (OD) was read at 450 nm (Biotek, Synergy HT, VT). The percent of binding of sera blocked with wild type or mutated peptides to tau_2–18_ was calculated relative to the binding of sera without competing peptides to tau_2–18_ as 100%. The half maximal inhibitory concentration (IC_50_) for each peptide was calculated.

### Surface plasmon resonance (SPR)

Binding studies were performed at 25 °C using a Biacore 2000 optical biosensor equipped with a Protein A-coated sensor chip and equilibrated with running buffer (10 mM HEPES, 150 mM NaCl, 0.01% Tween-20, 0.1 mg/mL BSA, pH 7.4). The surfaces were regenerated with two 12-s injections of 150 mM phosphoric acid after each binding cycle.

Antibody captures for kinetic analysis of antigen binding. For each binding cycle, the antibodies were diluted into running buffer and injected across individual Protein A surfaces. These injections produced capture levels of ~ 55–75 RU (resonance units). Using a short-and-long injection approach, tau monomer and oligomer were tested in triplicate in a threefold dilution series starting at 40 nM (this concentration was established using an estimated average molecular weight of 138 kDa for the oligomer). For each tau/antibody interaction, the responses from the three runs were globally fitted to a 1:1 interaction model (shown as the overlaid smooth red lines in the figures) to obtain the binding parameters listed in the table in Fig. 4.

### Detection of Aβ plaques and tau tangles in human brain tissues by IHC and confocal microscopy

Sera from mice immunized with AV-1959R/A, AV-1980R/A, and mixture of AV-1959R/A and AV-1980R/A, as well as control mice injected with Advax^CpG^ only, were screened for the ability to bind to human Aβ plaques or/and tau tangles using 40-μm brain sections of formalin-fixed cortical tissues from a severe AD case (received from Brain Bank and Tissue Repository, MIND, UC Irvine) using immunohistochemistry, as described previously [20–22]. In addition, brain sections were stained with anti-Aβ (beta-amyloid (1–42), 1:250, Invitrogen, CA) and humanized anti-tau (Armanezumab, 1:1000; Institute for Molecular Medicine, CA) antibody as positive controls. Sections were imaged using an Olympus FX1200 confocal microscope, with identical laser and detection settings across a given immunolabel.

### Mouse brain tissue preparation, immunohistochemistry, and confocal microscopy

Following perfusion, one hemisphere from each mouse was postfixed in 4% paraformaldehyde for 48 h then stored in PBS + 0.05% sodium azide. Fixed half-brains were placed in 30% sucrose for at least 48 h before being cut in the coronal plane (40-μm sections) using a freezing sliding microtome. Brain sections were rinsed in PBS before blocking in PBS + 0.05% Triton-X with 10% goat serum for 1 h. First, samples were stained with Amylo-Glo™ RTD Amyloid Plaque Stain Reagent (Biosensis, Australia) for 15 min, washed three times, and then incubated in pS199 (Abcam, UK, 1:1000) and PHF-1 (gift from Dr. Peter Davis, 1:1000) phospho-tau primary antibodies at 4 °C overnight. The next day, sections were washed three times with PBS and placed in appropriate Alexa Fluor-conjugated secondary antibody solutions at room temperature for 1 h. Sections were rinsed three additional times, mounted onto slides, and coverslipped using Fluoromount-G. For confocal microscopy, immunofluorescent staining was performed on equivalent brain sections and imaged on the Olympus FX1200 confocal microscope. Tau tangles and β-amyloid plaques were visualized using Z-stack maximum-projection images taken through the entire depth of the section at 1-μm intervals.

### Biochemical analyses

Right hemispheres, previously frozen on dry ice and stored at − 80 °C, were crushed on dry ice using mortar and pestle, then homogenized in solution of T-PER (Thermo Scientific, Waltham, MA) and phosphatase and protease inhibitor mixtures (Thermo Scientific, MA and Roche, CA) and processed as previously described [22–24]. Quantitative biochemical analysis of human Aβ was conducted using commercially available electrochemiluminescent multiplex assay system [Meso Scale Discovery (MSD)]. Human Aβ triplex (6E10 capture antibody) was used for simultaneous measurement of Aβ38, Aβ40, and Aβ42 in both soluble and insoluble protein fractions [24].

Concentrations of human total and phosphorylated tau in samples (soluble and insoluble brain extracts) were determined by Tau (total) Human ELISA kit, Tau [pS396] Human ELISA Kit, Tau [pS199] Human ELISA Kit, Tau [pT181] Human ELISA Kit, and Tau [pT231] Human ELISA Kit (all from ThermoFisher Scientific, MA), according to the manufacturer’s instructions.

Soluble SDS-PAGE WB and quantifaction was performed following standard protocols as previously described [22–24]. Primary antibodies used for WB analysis included the following: Armanezumab (1:2000; Institute for Molecular Medicine, Huntington Beach, CA), anti-GFAP (1:500; Millipore-Sigma, MO), anti-P2RY12 (1:500; Millipore-Sigma, MO), and anti-CD45 (1:500; Bio-Rad, CA). All blot membranes were also labeled with anti-β-actin antibodies (1:1000; Millipore-Sigma, MO) as a loading control.

### Statistical analysis

All statistical parameters [mean, standard deviation (SD), significant difference, etc.] used in experiments were calculated using Prism 6 software (GraphPad Software, Inc.). Statistically significant differences were examined using unpaired *t* test or one-way ANOVA with Tukey’s multiple comparisons test (*p* value < 0.05 was considered as statistically different).

## Results

### Immunogenicity of vaccines in T5x mice

**Fig. 2:**
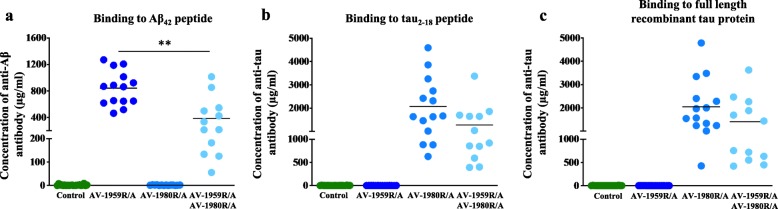
MultiTEP-based vaccines induced high titers of anti-Aβ and anti-tau antibodies in T5x mice. Concentration of antibodies binding to Aβ_42_ (**a**), tau_2–18_ peptide (**b**), and full-length recombinant tau protein (**c**) was detected in sera of male and female mice by ELISA. Lines represent the average value for combined male and female mice (***p ≤ 0.001). Of note, mice immunized with AV-1959R/A did not induce antibodies specific to tau, while vaccination of mice with AV1980R/A did not generate ant-Aβ antibodies

We previously found that a MultiTEP vaccine carrying both Aβ and tau epitopes together within a single protein, AV-1953R, induced significantly lower titers of anti-tau antibodies in wildtype mice compared with combined delivery of two separate anti-Aβ, AV-1959R, and anti-tau, AV-1980R vaccines [10]. Therefore, to test the efficacy of combination therapy, we instead here used a mixture of two vaccines: (1) AV-1959R carrying three copies of Aβ B cell epitope (Aβ_1–11_) attached to MultiTEP and (2) AV-1980R, carrying three copies of tau N-terminal epitope (tau_2–18_) attached to MultiTEP, formulated in Advax^CpG^ adjuvant (AV-1959R/A + AV-1980R/A). Two-month-old T5x bigenic mice were immunized with the mixture of both vaccines or with each vaccine separately (Fig. 1). Mice in all groups generated high titers of antibodies specific to Aβ_42_ and/or tau_2–18_ (Fig. 2, Additional file 1: Figure S1). The humoral response in all groups was high, ranging from 55 to 4785 μg/ml; however, the average concentrations of anti-Aβ (Fig. 2a) antibodies were significantly lower in mice immunized with the AV-1959R/A + AV-1980R/A combination compared to mice vaccinated with AV-1959R/A vaccine alone. Similar, but non-significant trend was observed in mice immunized with AV-1980R/A compared with combination vaccines (Fig. 2b). In addition, the antibodies obtained bound equally well in ELISA to both the tau_2–18_ peptide and the full-length recombinant tau protein (Fig. 2b, c). As expected, these vaccines were specific, so immunization with AV-1980R/A alone did not generate anti-Aβ antibodies and immunization with AV-1959R/A alone did not induce production of anti-tau antibodies **(**Fig. 2**).**

**Fig. 3:**
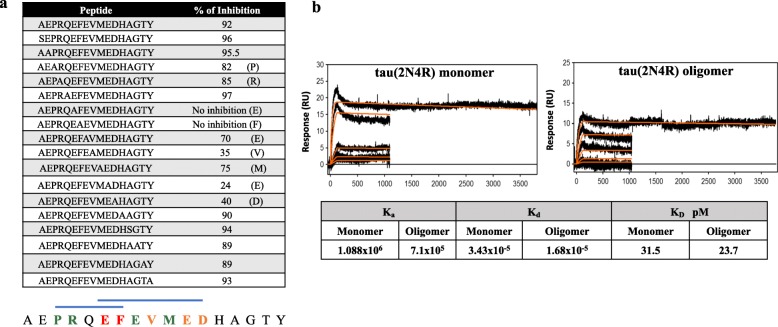
**a** Epitope mapping of immune sera was performed by alanine scanning competition ELISA. Two overlapped epitopes have been detected comprising amino acids 4–9 PRQEFE and amino acids 7–13 EFEVMED. Percent of inhibition of antibody binding to Tau_2–18_ peptide with mutated peptides (alanine substitution of each single amino acid) is shown in the table. **b** Binding avidity of anti-tau_2–18_ antibodies generated in T5x mice was determined by surface plasmon resonance (SPR). Monomeric and oligomeric forms of recombinant human tau (2N4R) were passed through antibodies immobilized on a Protein A-coated sensor chip

Previously, we mapped the epitope for monoclonal antibody generated after immunization of mice with AV-1980R/A [16]. Here, we also mapped the epitopes recognized by antibodies induced in mice vaccinated with AV-1980R/A using alanine scanning. The data demonstrated that in bigenic mice, AV-1980R/A active vaccination generated antibodies specific to two overlapping epitopes comprising amino acids 4–8 (PRQEFE) and 7–13 (EFEVMED) of the N-terminus of human tau (Fig. 3a). The binding avidity of anti-Aβ_11_ polyclonal antibodies generated by AV-1959R/A vaccination in mice [25], rabbits [26], and monkeys [14] to different forms of Aβ_42_ (monomeric, oligomeric, and fibrillar) was previously demonstrated. Thus, in this study, we sought to measure the binding avidity of anti-tau polyclonal antibodies purified from sera of AV-1980R/A vaccinated T5x mice to monomeric and oligomeric forms of recombinant Tau (2N4R) protein. As shown in Fig. 3b, immobilized anti-tau antibodies bound to tau monomers and oligomers with high avidity (KD = 31.5pM for monomers and 23.7pM for oligomers), indicating a marginally higher avidity of the antibodies for tau oligomers than monomers.

**Fig. 4:**
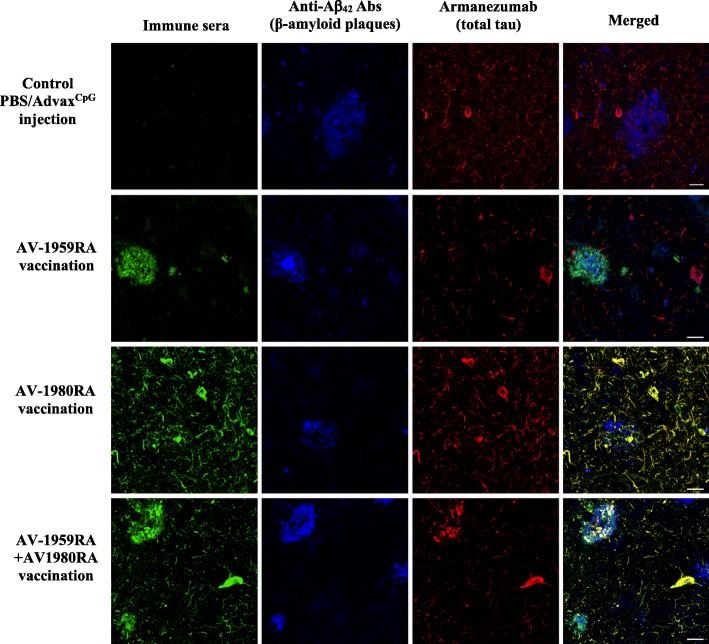
AV-1959R/A and AV-1980R/A immune sera (both separately and mixture), but not Advax^CpG^ injected (control) sera bound to the 50-μm brain sections of cortical tissues from an AD case, female 89 years old, Plaque stage-C, Tangle stage-6. Brain sections were stained with anti-Aβ (beta-amyloid (1–42), 1:250) and humanized anti-tau (Armanezumab, 1:1000) antibody as positive controls (scale = 20 μm)

Next, we examined whether the immune mouse sera could recognize human senile plaques and tau-laden neurofibrillary tangles, by performing immunofluorescent staining of brain sections of AD case. As expected, sera from T5x mice immunized with AV-1959R/A bound only to amyloid plaque pathology. Conversely, sera from AV-1980R/A-vaccinated mice bound to tau tangles (NFT) and neuritic threads, but not amyloid plaques. Importantly, immune sera from mice vaccinated with the combined vaccines labeled both hallmark pathologies: amyloid plaques and neurofibrillary tangles (Fig. 4). The specific binding of sera (green) to amyloid plaques (blue) and/or NFTs and neuritic threads (red) was further demonstrated by co-labeling brain sections with anti-Aβ_42_ Ab, a marker of beta-amyloid, and humanized Armanezumab mAb that is specific to the N-terminal epitope of Tau. Importantly, no binding of AD brains was observed with sera from control mice injected with Advax^CpG^ adjuvant only. Thus, combination therapy with the mixture of MultiTEP-platform-based vaccines can elicit a strong epitope-specific antibody response targeting simultaneously both of the misfolded proteins involved in AD pathology.

### Changes in Aβ and tau pathology in the brains of T5x mice immunized with single or dual vaccines

Previously, we showed that bigenic T5x mice exhibit accelerated tau pathology compared with the parental THY-Tau22 strain and reduced insoluble Aβ_42_ compared with parental 5XFAD mice [11]. We analyzed changes in both soluble and insoluble forms of these proteins in brain homogenates from vaccinated mice using sensitive ELISAs for total tau and tau phosphorylated at positions pS199, pT231, pS396, and pT181 and by Meso Scale Discovery (MSD) analysis of Aβ_42_, Aβ_40_, and Aβ_38_.

**Fig. 5:**
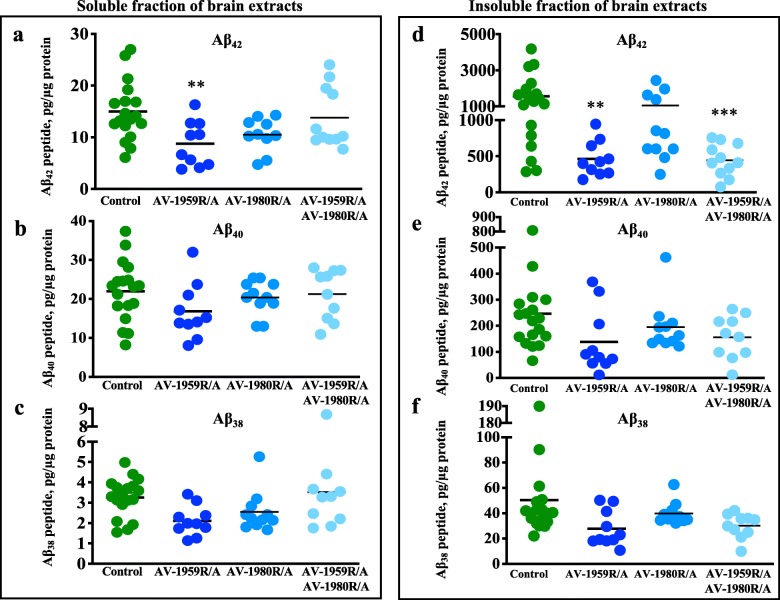
Effect of protein vaccination on Aβ proteins in T5x mice. Level of human Aβ_42_ (**a**, **d**), Aβ_40_ (**b**, **e**), and Aβ_38_ (**c**, **f**) peptides in brain soluble (**a**–**c**) and insoluble (**d**–**f**) extractions were analyzed by MSD assay. Lines represent average (*p ≤ 0.05; **p ≤ 0.01)

Using this approach, we detected a significant reduction in both soluble and insoluble levels of Aβ_42_ in mice immunized with AV-1959R/A (Fig. 5a, d). Mice immunized with a combination vaccine showed a significant reduction of insoluble Aβ_42_ (Fig. 5d). However, when these cohorts were analyzed by gender, we observed a significant reduction of soluble Aβ_42_ in female mice received mixed vaccine, but not AV-1959R/A alone (Additional file 1: Figure S2a), while male mice showed significant reduction of soluble Aβ_42_ in AV-1959R/A cohort and only a slight trend of reduction in AV-1959R/1980R/A cohort (Additional file 1: Figure S3a), thus making this difference non-significant when combined female/male groups were compared (Fig. 5a**)**. In contrast, insoluble amyloid-β was significantly reduced in male mice (Additional file 1: Figure S3d) and showed a well-pronounced downward trend in female mice (Additional file 1: Figure S2d). These data may reflect the sex-dependent impact of antibodies on Aβ pathology. A non-significant trend towards decreased Aβ_40_ (Fig. 5b, e) and Aβ_38_ (Fig. 5c, f) was also observed in mice immunized with AV-1959R/A.

**Fig. 6:**
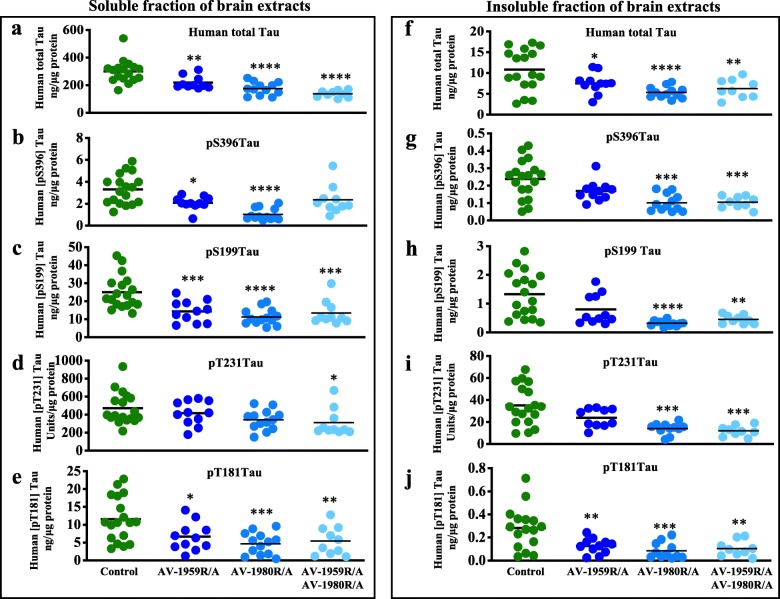
Effect of protein vaccination on tau proteins in T5x mice. Level of human total tau protein (**a**, **f**) and several phosphorylated tau species (**b**–**e** and **g**–**j**) in brain soluble (**a**–**e**) and insoluble (**f**–**j**) extractions were analyzed by ELISA. Lines represent average (*p ≤ 0.05; **p ≤ 0.01; ***p ≤ 0.001; ****p ≤ 0.0001)

Significant reductions in total and phosphorylated soluble tau were seen in mice treated with the tau vaccine AV-1980R/A as well as mice treated with the vaccine combination, AV-1959R/A + AV-1980R/A (Fig. 6a–e). Interestingly, we also detected significant reductions of several phosphorylated species of tau in mice immunized with the β-amyloid vaccine, AV-1959R/A, alone (Fig. 6a–c; Additional file 1: Figures S4 and S5), indicating that decreases in Aβ_42_ also led to a decrease of tau pathology, consistent with previous reports in the 3xTg-AD mouse model [27]. The pattern of reduced species of soluble Tau was slightly different in female and male mice immunized with either anti-Aβ or anti-Tau vaccines alone or in combination (Additional file 1: Figures S4 and S5). These differences could be associated with differences in the pathological burden of Aβ and Tau in mice of different genders as well as the titers of induced antibodies. Female mice have higher levels of Aβ_42_, but lower levels of Tau than male mice. Additional studies that employ greater numbers of both male and female mice will be needed to fully characterize the potential differential changes that occur between male and female immunized T5x mice. Regardless of these intriguing sex-dependent differences, the reduction of all analyzed soluble tau species in the combined gender groups demonstrates the robust effect on multiple tau species achieved by this approach.

Analysis of insoluble species of Aβ and Tau also revealed sex-dependent effects of vaccination. Although vaccinated female mice showed a downward trend in both Aβ_42_ and tau molecules, a significant reduction was observed only in male mice (Additional file 1: Figure S4f-g; Figure S5f-j) leading to significant decrease in the combined male and female groups for all detected insoluble tau species (Fig. 6f–j and Additional file 1: Figure S4e).

To determine whether immunization might lead to changes in microgliosis and astrocytosis, we performed WB with antibodies specific to GFAP, P2RY12, and CD45 (Additional file 1: Figure S6). Somewhat suprisingly, no differences were detected in the expression of the astrocytic activation marker GFAP, the microglial homoeostatic marker P2RY12, or the myeloid activation marker CD45, between control and vaccinated T5x mice. These data suggest that although antibody-mediated reduction in pathology can be expected to decrease glial activation, beta-amyloid and tau antibodies may alternatively offset any such response by activating microglia via Fc-mediated signaling. Thus, these potentially apposing effects might result in no significant changes in glial activation.

As ELISA measurements provide a more quantitative approach than histology, we used ELISA to measure soluble and insoluble Aβ, tau, and phospho-tau species to determine the effectiveness of the AV-1959R/A, AV-1980R/A, and combined vaccination approach. However, we also asked whether an immunohistochemical and confocal microscopy approach might provide a further indication of altered pathology. Sections from 3 mice per group were examined using double-labeling for PHF-1 and pS199 tau along with Amylo-Glo, a Thioflavin S analog that labels the beta-pleated sheet confirmation of amyloid plaques. Confocal Z-stacks were captured, with representative images (Additional file 1: Figure S7) indicating that AV-1959R/A vaccination or the combined AV-1959R/A + AV-1980R/A approach successfully reduced both Aβ and tau pathology. In contrast, vaccination with AV-1980R/A alone reduced Tau pathology but not Aβ plaques, as would be expected from the biochemical data detailed above (Fig. 5).

## Discussion

Despite some encouraging clinical data for BAN2401 monoclonal antibody (mAb), which selectively binds large soluble Aβ protofibrils [28], the vast majority of Aβ therapeutics trials, including but not limited to active and passive vaccines, have thus far failed to modify cognition in MCI and AD subjects [29–31]. The failure of these trials may reflect treatment being initiated too late in the pathological process when neuronal damage or other downstream pathologies cannot be reversed. Therefore, thinking has moved to the idea that Aβ-based mono treatment should be initiated as a preventive rather than a therapeutic measure. Such a long-term prophylactic strategy could likely only be practically achieved in terms of both compliance and affordability, through the use of a safe and immunogenic vaccine rather than monoclonal antibody infusions [28, 30, 32–38]. Nevertheless, based on the recently published data indicating that Aβ and tau aggregates interact synergistically to drive downstream neurodegeneration [1, 2, 9, 39, 40], we hypothesize that vaccines targeting both pathological molecules simultaneously might be the most effective therapeutic approach [10].

The current study tested the immunogenicity of AV-1959R/A and AV-1980R/A as a combined vaccine in the T5x bigenic mouse model of AD which exhibits robust accumulation of intraneuronal tau and extensive extracellular amyloid plaque pathology within the hippocampus, neocortex, and amygdala by 7 months of age [11]. By comparison with wildtype mice [10], humoral immune responses in bigenic mice vaccinated either with AV-1959R/A or with AV-1980R/A alone were higher than in mice vaccinated with combined vaccines (Fig. 2 and Additional file 1: Figure S1). Notably, combined vaccines formulated in Advax^CpG^ adjuvant successfully generated high concentrations (55 to 4785 μg/ml) of antibodies specific to both Aβ and tau (Fig. 2 and Additional file 1: Figure S1). These milligram levels of antibodies in the sera of vaccinated animals are due to the MultiTEP vaccine platform that strongly activates T helper cells in mice of H-2^b^ immune haplotype [10, 41]. Importantly, this universal MultiTEP vaccine platform is designed to provide a broad coverage of human MHC class II polymorphism by utilizing a wide array of tetanus toxin, hepatitis B, and influenza Th epitopes incorporated into the MultiTEP platform [26]. These foreign Th epitopes incorporated into the MultiTEP platform are very immunogenic in mice of different haplotypes, in rabbits, and in monkeys with highly polymorphic MHC class II genes [14, 22, 26, 41, 42]. A growing amount of evidence suggests that prophylactic vaccination delivered prior to clinical symptoms may be needed to prevent the development of AD fully. However, therapeutic treatments that target amyloid and tau pathology and alleviate key symptoms and/or slow the progression of AD are still desperately needed to treat the millions of people currently affected by AD. Yet, it is well known that older people respond poorly to new vaccines due to immunosenescence, characterized by an abundance of memory T cells and a decrease in the number of naive T cells with age [43]. The MultiTEP strategy provides a unique opportunity to generate high levels of antibodies in the elderly by activating not only naïve Th cells, but also pre-existing memory Th cells previously generated in response to infections and/or vaccinations with tetanus toxin, hepatitis B, and influenza, thereby overcoming immunosenescence. Indeed, the feasibility of this strategy based on pre-existing memory Th cells was previously demonstrated [10, 21, 44].

Earlier, we demonstrated that in mice and monkeys, AV-1959 induced antibodies specific to the AEFRH epitope of Aβ_1–11_ peptide incorporated in this vaccine [41]. This immunodominant B cell epitope of N-terminus of Aβ_42_ [45] is widely used in preclinical and clinical studies, and data from various groups, including us, suggest that high-affinity antibodies specific to this region reduce AD-like pathology not only in mouse models of AD but also in brains of vaccinated people [18, 20, 21, 25, 44, 46–51]. Immunizations with AV-1959R/A also induced therapeutically potent antibodies that significantly reduced soluble and insoluble Aβ_42_ pathology in this pathologically aggressive bigenic mouse model of AD (Fig. 5).

Here, we also mapped the immunogenic B cell epitopes of tau_2–18_ and demonstrated that antibodies induced by AV-1980R/A recognized two overlapping epitopes comprising 4–8 aa and 7–13 aa (Fig. 3). The later epitope coincides with that of the TNT1 mAb, which is shown to recognize pre-tangle pathology in early Braak stages and more compact classical neurofibrillary tangles, but not late-stage ghost tangles [17]. We previously compared the binding of Armanezumab, mAb specific to aa 4–8, with TNT1 and showed that, under denaturing conditions, both antibodies bind to tau protein in AD, but not in non-AD brains. However, under denaturing conditions, Armanezumab recognizes tau aggregates with a higher molecular weight compared to TNT1 [16]. Thus, we believe that AV-1980R/A, which induces antibodies specific to two B cell epitopes, might be therapeutically more effective than the mAb. In fact, in the stringent T5x bigenic mouse model of AD used here, AV-1980R/A vaccine has induced high-affinity antibodies (Fig. 3) that significantly decreased soluble and insoluble total and various phosphorylated tau species in the brains of vaccinated animals (Fig. 6).

We expected that neuropathological changes in the brains of mice immunized with a combination vaccine would be more pronounced compared to single vaccinated mice, but the reduction in Aβ_42_ and various tau species was comparable in mice immunized with either a single or a combination vaccine. Perhaps this can be explained by data showing that the combined vaccine generated lower titers of anti-Aβ and anti-tau antibodies in mice than single vaccines (Fig. 2). Nevertheless, we anticipate that a simultaneous reduction of both pathological molecules may lead to a better improvement in cognitive functions, although future studies will need to carefully test this question in appropriate mouse models. Such behavioral studies may need to be tested in a less aggressive mouse model that better reflects the more common, sporadic form of Alzheimer’s disease in which the time window for effective prevention and treatment is likely wider, e.g., APP knock-in mice crossbred with humanized Tau knock-in mice [52–57]. As cognitive deficits tend to develop at later ages in knock-in models, these mice likely represent models of preclinical AD that could be well suited for testing preventative active vaccines.

Interestingly, we observed a significant decrease of tau in mice immunized with a single vaccine targeting Aβ, AV-1959R/A (Fig. 6), but did not see decreased Aβ_42_ in mice immunized with single anti-tau vaccine, AV-1980R/A (Fig. 5). This data, as well as pervious data showing a threefold increase in misfolded and hyperphosphorylated tau in mice generated by crossing of Thy-Tau22 with 5xFAD mice [11] further, supports the hypothesis that Aβ can accelerate and exacerbate tau pathology.

## Conclusions

Here, we showed that combined active vaccine based on the MultiTEP platform and formulated with Advax^CpG^ adjuvant, which has been shown to be safe and effective in human trials [58–61], is highly immunogenic in bigenic mice exhibiting both Aβ and tau pathologies. Generated antibodies specifically recognize Aβ plaques, neurofibrillary tangles, and neuritic threads in human AD tissue, and most importantly, vaccination leads to significant decreases in multiple soluble and insoluble tau species and insoluble Aβ_42_ in the brains of transgenic mice. This data suggests that a combined vaccination approach could potentially be used to induce strong immune responses against both of the hallmark pathologies of AD in a broad population of vaccinated subjects with high MHC class II gene polymorphisms.

## Supplementary information

**Additional file 1: Figure S1.** Humoral immune responses in female and male T5x mice vaccinated with different vaccines. Concentration of antibodies binding to Aβ42 (a, d), tau2-18 peptide (b, e) and full-length recombinant tau protein (c, f) were detected in sera by ELISA. Lines represent average. Statistically significant differences were examined using unpaired *t-test* (**p ≤ 0.01; Control group - n=8 for female and n=14 for male; AV-1959R/A group - n=6 for female and n=8 for male, AV-1980R/A group - n=6 for female and n=8 for male and AV-1959R/A+AV-1980RA group - n=5 for female and n=7 for male). **Figure S2.** Effect of protein vaccination on Aβ proteins in female, T5x mice. Level of human Aβ42 (a and d), Aβ40 (b and e) and Aβ38 (c and f) peptides in brain soluble (a-c) and insoluble (d-f) extractions were analyzed by MSD assay. Error bars represent average ± SEM. Statistically significance were calculated against Control group using ANOVA test (*p ≤ 0.01; Control group - n=8 for female and n=14 for male; AV-1959R/A group - n=6 for female and n=8 for male, AV-1980R/A group - n=6 for female and n=8 for male and AV-1959R/A+AV-1980RA group - n=5 for female and n=7 for male). **Figure S3.** Effect of protein vaccination on Aβ proteins in male, T5x mice. Level of human Aβ42 (a and d), Aβ40 (b and e) and Aβ38 (c and f) peptides in brain soluble (a-c) and insoluble (d-f) extractions were analyzed by MSD assay. Error bars represent average ± SEM. Statistically significance were calculated against Control group using ANOVA test (*p ≤ 0.05; ***p ≤ 0.001; Control group - n=8 for female and n=14 for male; AV-1959R/A group - n=6 for female and n=8 for male, AV-1980R/A group - n=6 for female and n=8 for male and AV-1959R/A+AV-1980RA group - n=5 for female and n=7 for male). **Figure S4.** Effect of protein vaccination on tau proteins in female, T5x mice. Level of human total tau protein (a, f) and several phosphorylated tau species (b-e and g-j) in brain soluble (a-e) and insoluble (fj) extractions were analyzed by ELISA. Error bars represent average ± SEM. Statistically significance were calculated against Control group using ANOVA test (*p ≤ 0.05; **p ≤ 0.01; ***p ≤ 0.001; ****p ≤ 0.0001; Control group - n=8 for female and n=14 for male; AV-1959R/A group - n=6 for female and n=8 for male, AV-1980R/A group - n=6 for female and n=8 for male and AV-1959R/A+AV-1980RA group - n=5 for female and n=7 for male). **Figure S5.** Effect of protein vaccination on tau proteins in male, T5x mice. Level of human total tau protein (a, f) and several phosphorylated tau species (b-e and g-j) in brain soluble (a-e) and insoluble (fj) extractions were analyzed by ELISA. Error bars represent average ± SEM. Statistically significance were calculated against Control group using ANOVA test (*p ≤ 0.05; **p ≤ 0.01; ***p ≤ 0.001; ****p ≤ 0.0001; Control group - n=8 for female and n=14 for male; AV-1959R/A group - n=6 for female and n=8 for male, AV-1980R/A group - n=6 for female and n=8 for male and AV-1959R/A+AV-1980RA group - n=5 for female and n=7 for male). **Figure S6.** Vaccination with protein vaccines did not change astrogliosis and microgliosis in brains of T5x mice. The levels of GFAP, P2RY12 and CD45 proteins in the soluble fraction of the brain extracts were analyzed by Western blotting and quantitatively determined by densitometric analysis with normalization against β-actin. The relative protein level in the brains of vaccinated mice is presented as a percentage of the protein level in the brains of control mice. Error bars represent average ± SEM. Statistically significant differences were examined using one-way ANOVA (n = 12 for Control group and n=11 for all vaccinated groups). **Figure S7.** Reduced β-amyloid and tau pathology in T5x mice following vaccination with different proteins. Representative pictures of brain CA1 region immunostained for Amylo-GloTM (ThS, anti-Aβ) and pS199 and PHF-1 (anti-tau) antibodies. Scale: 60μm (lowpwr) and 15μm (highpwr).

## Data Availability

All data generated or analyzed during this study are included in this published article and its supplementary information files.

## Abbreviations

AD: Alzheimer disease
Aβ: β-Amyloid
PHF: Paired helical filaments
T5x: Tau22-5xFAD mice
ELISA: Enzyme-linked immunosorbent assay
MSD: Meso Scale Discovery assay
PART: Primary age-related tauopathy
Th: T helper
NIA-AA: Alzheimer’s Association published revised guidelines
PET: Positron emission tomography
APP: Amyloid-β precursor protein
MCI: Mild Cognitive Impairment
ADNI: Alzheimer’s Disease Neuroimaging Initiative
SNAP: Suspected Non-Alzheimer Pathology
MHC: Major Histocompatibility Complex
HBV: Hepatitis B virus
TNT1: Tau N-terminal 1
*E. coli*: *Escherichia coli*
WB: Western blot
SPR: Surface plasmon resonance
PBS: Phosphate-buffered saline
HRP: Enzyme horseradish peroxidase
TMB: 3,3′,5,5′Tetramethylbenzidine
BSA: Bovine serum albumin
SD: Standard deviation
SEM: Standard error of the mean
