# Supplementary material for: Testing a MultiTEP-based combination vaccine to reduce Aβ and tau pathology in Tau22/5xFAD bigenic mice
Source: Alzheimers Res Ther. 2019 Dec 17;11:107. doi: 10.1186/s13195-019-0556-2 (PMC6918571; doi:10.1186/s13195-019-0556-2)
Supplement: Supplementary file 1 — Additional file 1: Figure S1. Humoral immune responses in female and male T5x mice vaccinated with different vaccines. Concentration of antibodies binding to Aβ42 (a, d), tau2-18 peptide (b, e) and full-length recombinant tau protein (c, f) were detected in sera by ELISA. Lines represent average. Statistically significant differences were examined using unpaired t-test (**p ≤ 0.01; Control group - n=8 for female and n=14 for male; AV-1959R/A group - n=6 for female and n=8 for male, AV-1980R/A group - n=6 for female and n=8 for male and AV-1959R/A+AV-1980RA group - n=5 for female and n=7 for male). Figure S2. Effect of protein vaccination on Aβ proteins in female, T5x mice. Level of human Aβ42 (a and d), Aβ40 (b and e) and Aβ38 (c and f) peptides in brain soluble (a-c) and insoluble (d-f) extractions were analyzed by MSD assay. Error bars represent average ± SEM. Statistically significance were calculated against Control group using ANOVA test (*p ≤ 0.01; Control group - n=8 for female and n=14 for male; AV-1959R/A group - n=6 for female and n=8 for male, AV-1980R/A group - n=6 for female and n=8 for male and AV-1959R/A+AV-1980RA group - n=5 for female and n=7 for male). Figure S3. Effect of protein vaccination on Aβ proteins in male, T5x mice. Level of human Aβ42 (a and d), Aβ40 (b and e) and Aβ38 (c and f) peptides in brain soluble (a-c) and insoluble (d-f) extractions were analyzed by MSD assay. Error bars represent average ± SEM. Statistically significance were calculated against Control group using ANOVA test (*p ≤ 0.05; ***p ≤ 0.001; Control group - n=8 for female and n=14 for male; AV-1959R/A group - n=6 for female and n=8 for male, AV-1980R/A group - n=6 for female and n=8 for male and AV-1959R/A+AV-1980RA group - n=5 for female and n=7 for male). Figure S4. Effect of protein vaccination on tau proteins in female, T5x mice. Level of human total tau protein (a, f) and several phosphorylated tau species (b-e and g-j) in brain soluble (a-e) and insoluble (fj) ex [file 13195_2019_556_MOESM1_ESM.pdf]

Testing a MultiTEP-based combination vaccine to reduce Aβ and tau pathology in Tau22/5xFAD bigenic mice

Hayk Davtyan, Armine Hovakimyan, Sepideh Kiani Shabestari, Tatevik Antonyan, Morgan A. Coburn, Karen Zagorski, Gor Chailyan, Irina Petrushina, Olga Svystun, Emma Danhash, Nikolai Petrovsky, David H. Cribbs, Michael G. Agadjanyan, Mathew Blurton-Jones, Anahit Ghochikyan

Supplementary Information

Figure S1

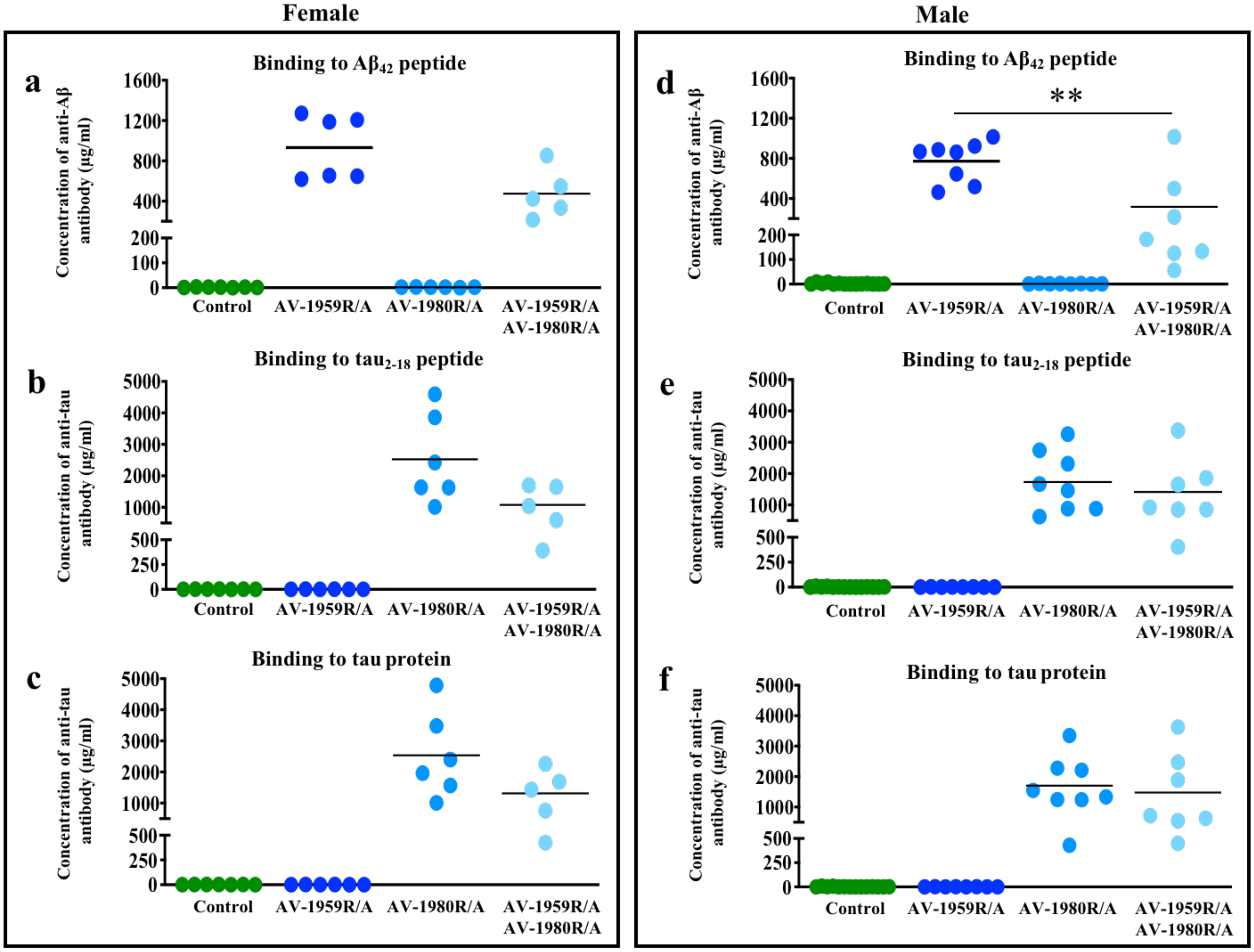

Figure S1. Humoral immune responses in female and male T5x mice vaccinated with different vaccines. Concentration of antibodies binding to Aβ_{42} (**a, d**), tau$_{2\text{-}18}$ peptide (**b, e**) and full-length recombinant tau protein (**c, f**) were detected in sera by ELISA. Lines represent average. Statistically significant differences were examined using unpaired *t-test* (*$**p \leq 0.01$;* Control group - n=8 for female and n=14 for male; AV-1959R/A group - n=6 for female and n=8 for male, AV-1980R/A group - n=6 for female and n=8 for male and AV-1959R/A+AV-1980RA group - n=5 for female and n=7 for male).

Figure S2

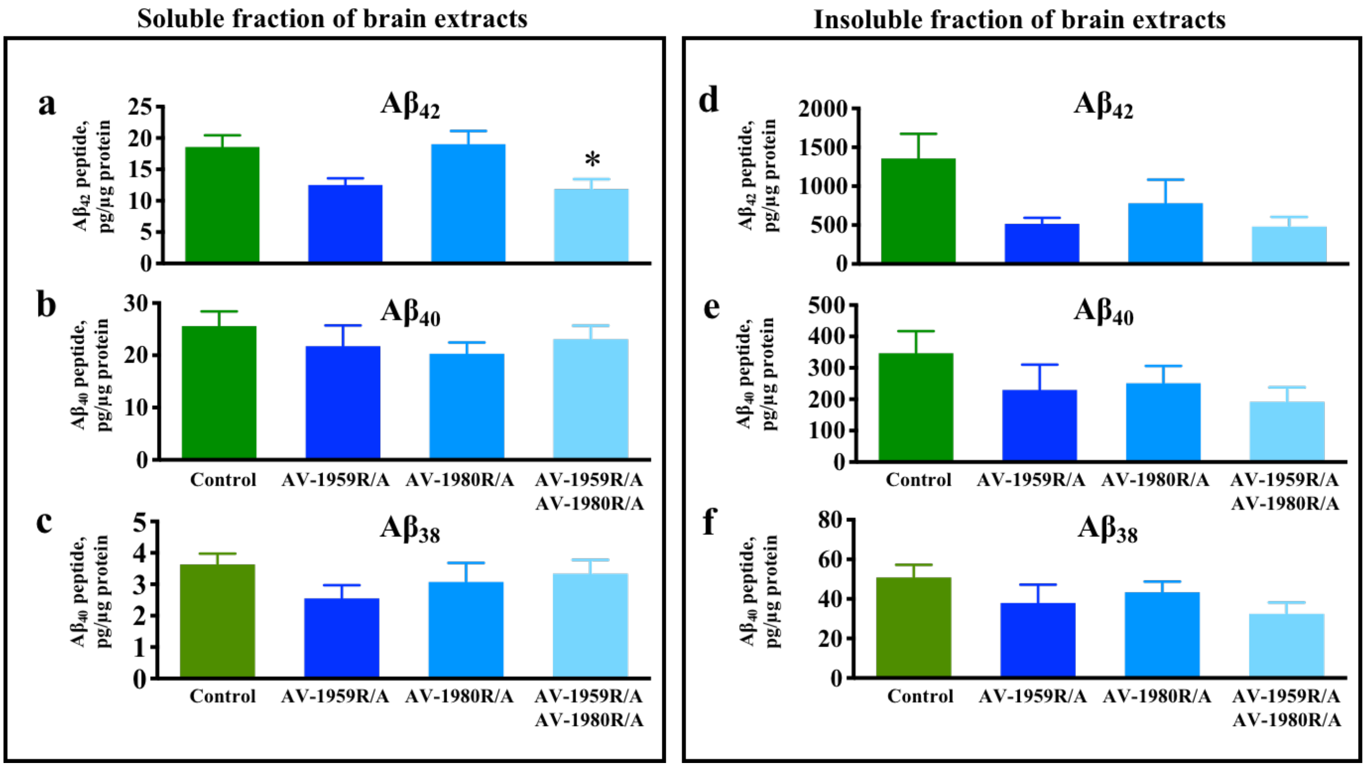

Figure S2. Effect of protein vaccination on Aβ proteins in female, T5x mice. Level of human $A\beta_{42}$ (**a** and **d**), $A\beta_{40}$ (**b** and **e**) and $A\beta_{38}$ (**c** and **f**) peptides in brain soluble (**a-c**) and insoluble (**d-f**) extractions were analyzed by MSD assay. Error bars represent average ± SEM. Statistically significance were calculated against Control group using ANOVA test (*$*p \leq 0.01$;* Control group - n=8 for female and n=14 for male; AV-1959R/A group - n=6 for female and n=8 for male, AV-1980R/A group - n=6 for female and n=8 for male and AV-1959R/A+AV-1980RA group - n=5 for female and n=7 for male).

Figure S3

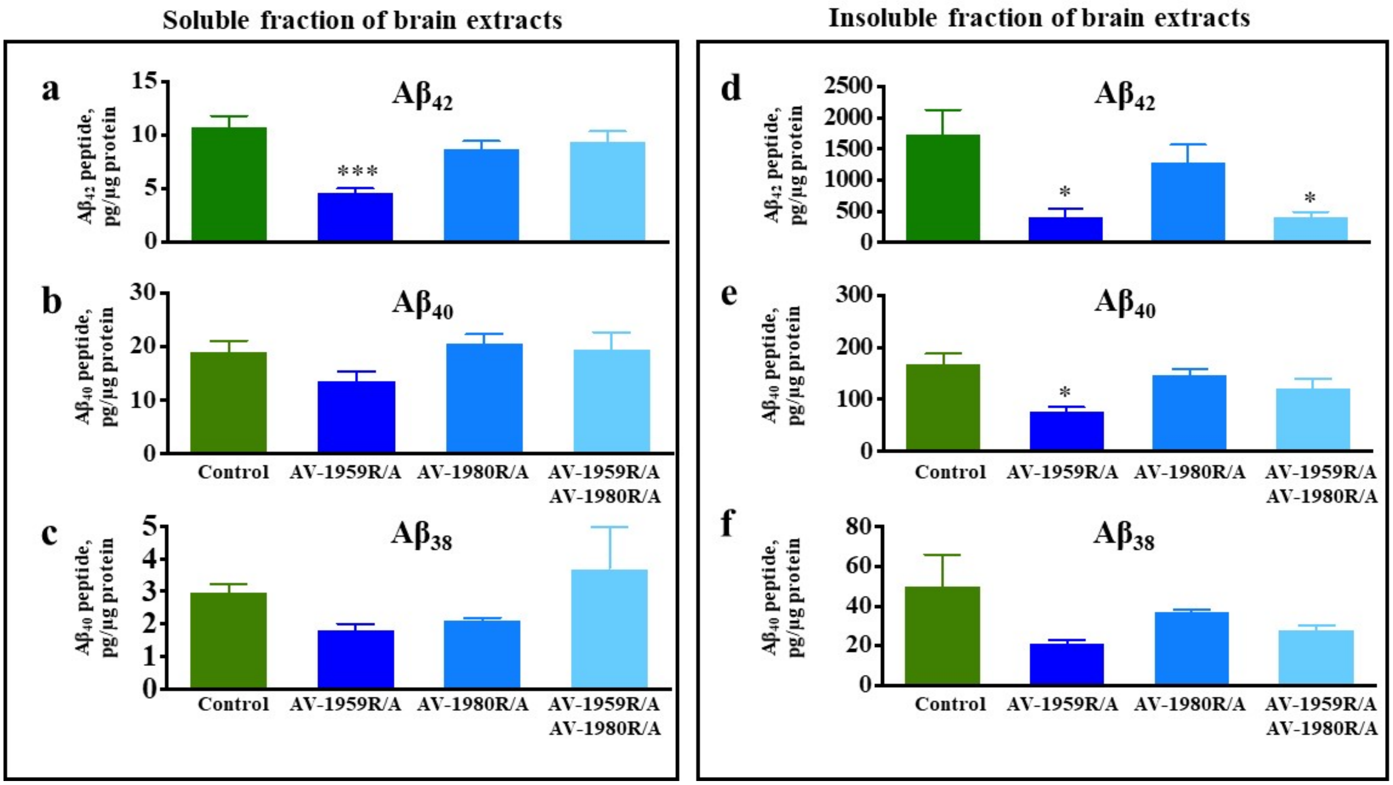

Figure S3. Effect of protein vaccination on Aβ proteins in male, T5x mice. Level of human $A\beta_{42}$ (**a** and **d**), $A\beta_{40}$ (**b** and **e**) and $A\beta_{38}$ (**c** and **f**) peptides in brain soluble (**a-c**) and insoluble (**d-f**) extractions were analyzed by MSD assay. Error bars represent average ± SEM. Statistically significance were calculated against Control group using ANOVA test (*$*p \leq 0.05$; $***p \leq 0.001$;* Control group - n=8 for female and n=14 for male; AV-1959R/A group - n=6 for female and n=8 for male, AV-1980R/A group - n=6 for female and n=8 for male and AV-1959R/A+AV-1980RA group - n=5 for female and n=7 for male).

Figure S4

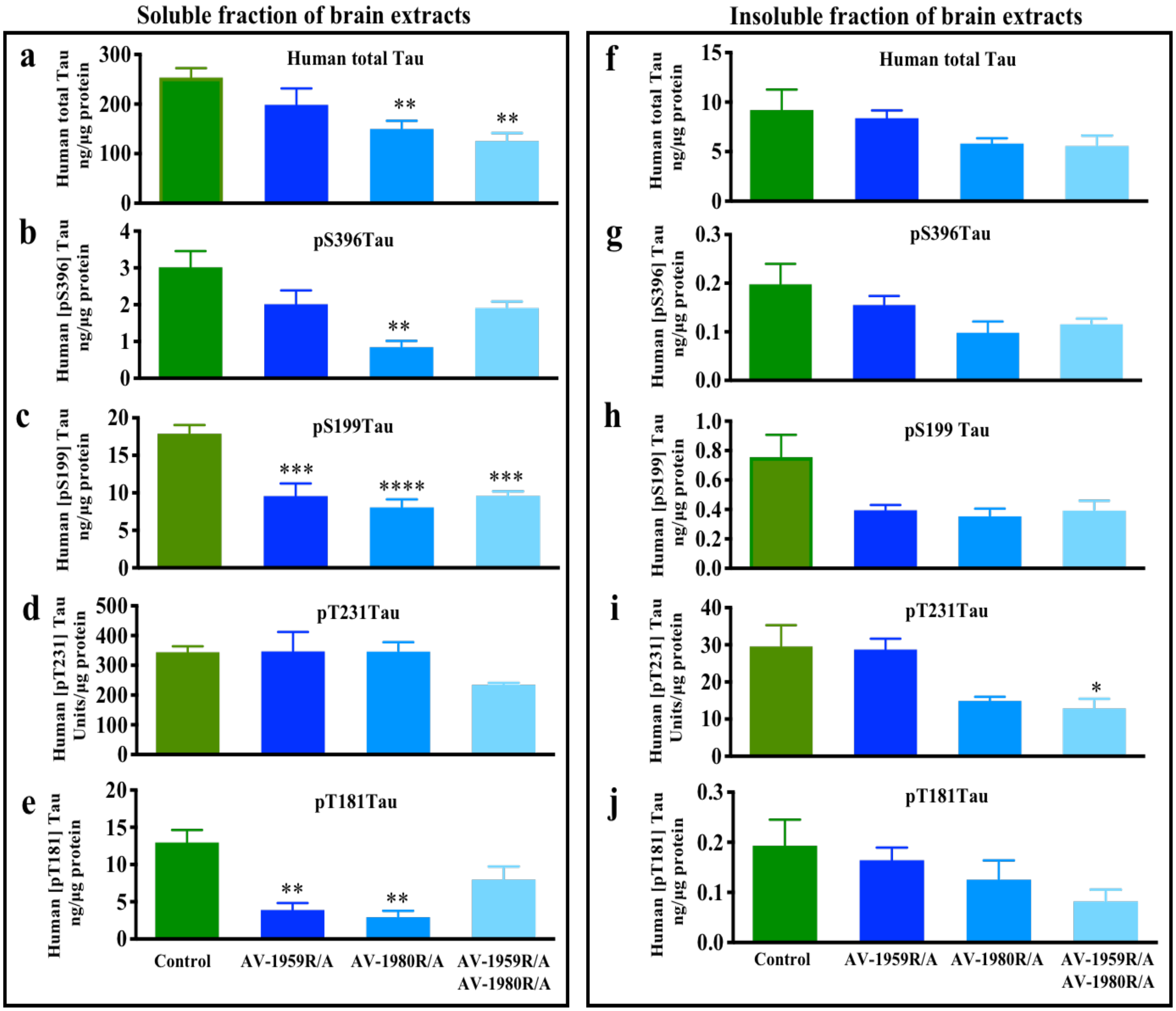

Figure S4. Effect of protein vaccination on tau proteins in female, T5x mice. Level of human total tau protein (**a, f**) and several phosphorylated tau species (**b-e** and **g-j**) in brain soluble (**a-e**) and insoluble (**f-j**) extractions were analyzed by ELISA. Error bars represent average ± SEM. Statistically significance were calculated against Control group using ANOVA test (*$*p \leq 0.05$; $**p \leq 0.01$; $***p \leq 0.001$; $****p \leq 0.0001$;* Control group - n=8 for female and n=14 for male; AV-1959R/A group - n=6 for female and n=8 for male, AV-1980R/A group - n=6 for female and n=8 for male and AV-1959R/A+AV-1980RA group - n=5 for female and n=7 for male).

Figure S5

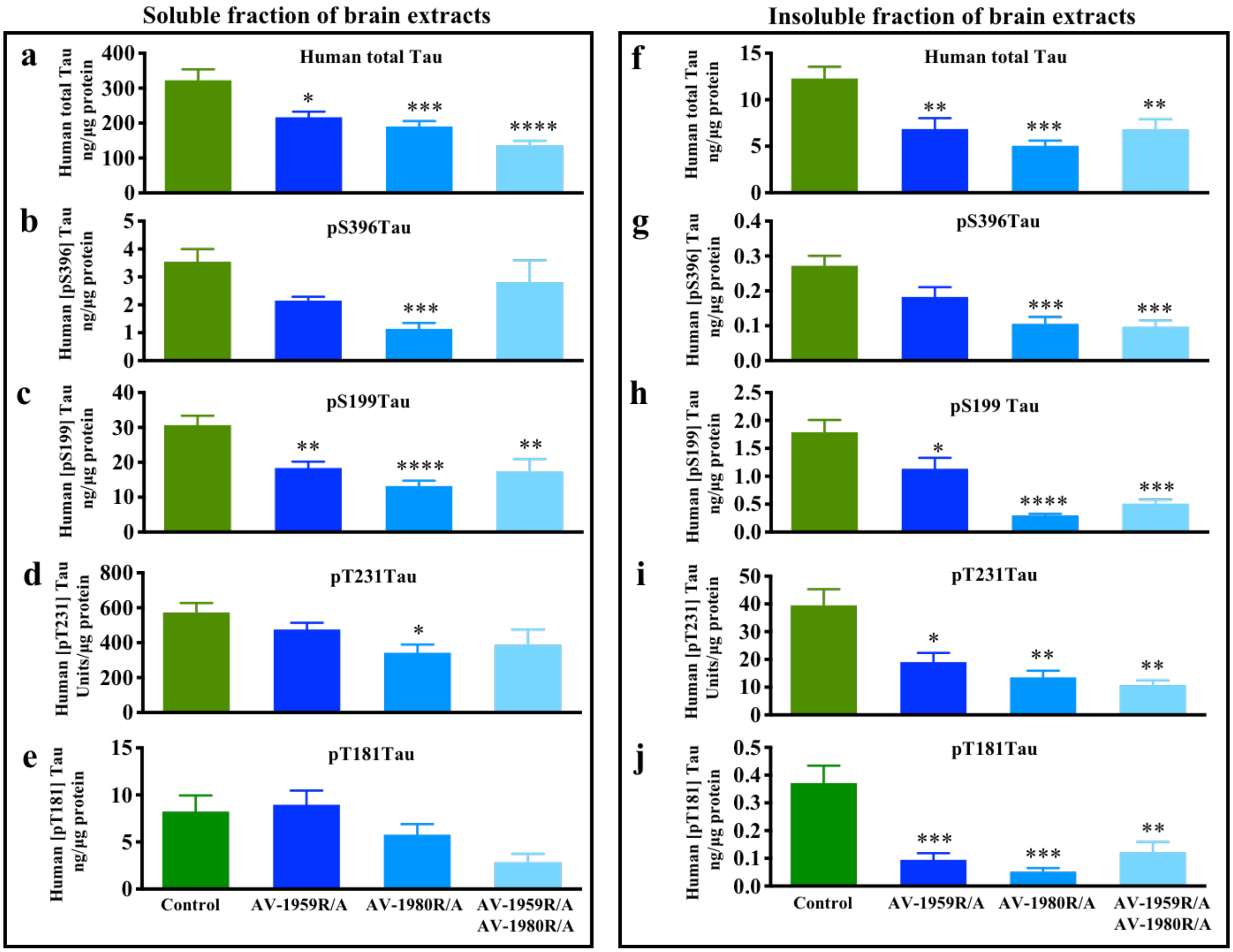

Figure S5. Effect of protein vaccination on tau proteins in male, T5x mice. Level of human total tau protein (**a, f**) and several phosphorylated tau species (**b-e** and **g-j**) in brain soluble (**a-e**) and insoluble (**f-j**) extractions were analyzed by ELISA. Error bars represent average ± SEM. Statistically significance were calculated against Control group using ANOVA test (*$*p \leq 0.05$; $**p \leq 0.01$; $***p \leq 0.001$; $****p \leq 0.0001$;* Control group - n=8 for female and n=14 for male; AV-1959R/A group - n=6 for female and n=8 for male, AV-1980R/A group - n=6 for female and n=8 for male and AV-1959R/A+AV-1980RA group - n=5 for female and n=7 for male).

Figure S6

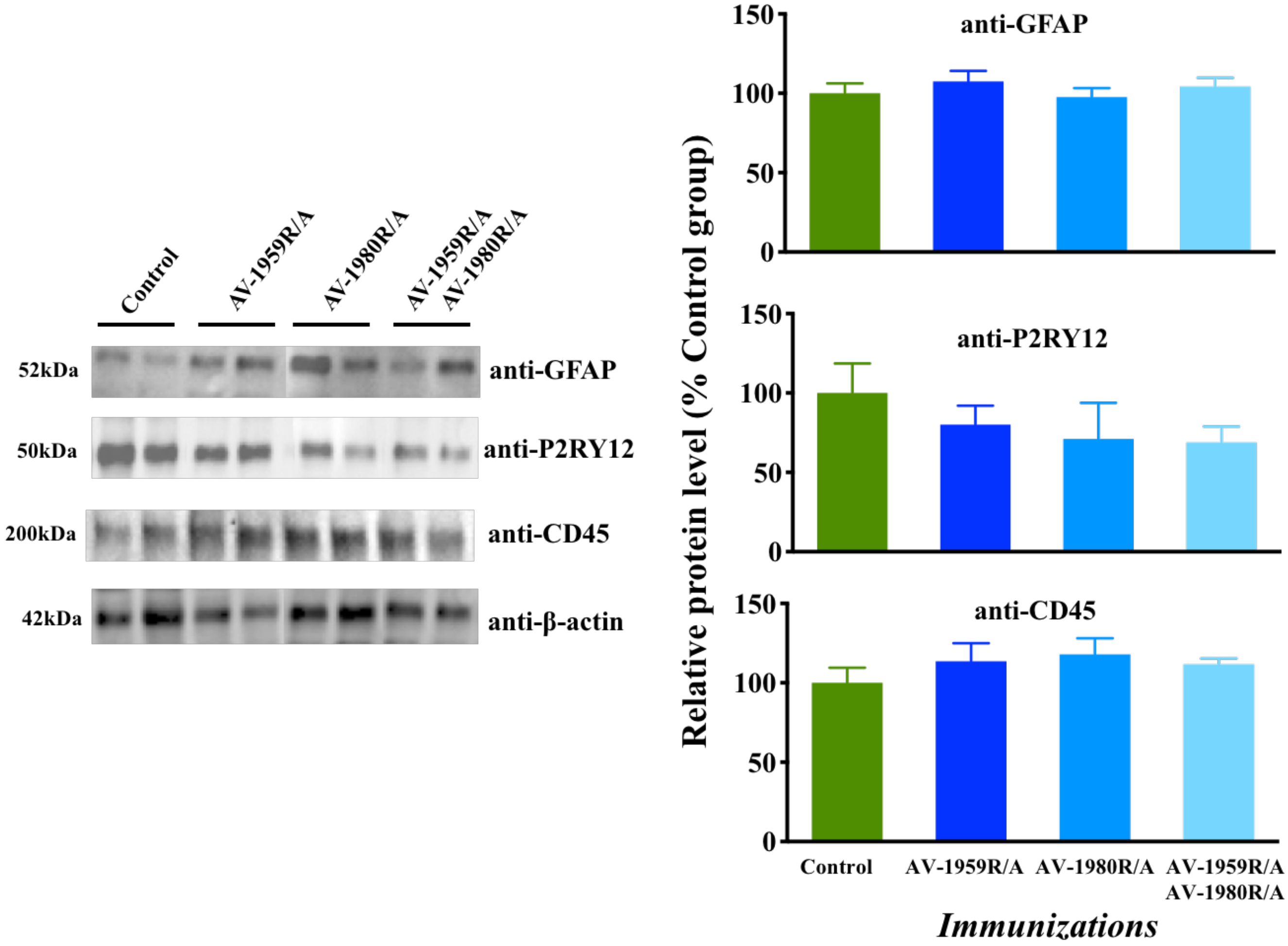

Figure S6. Vaccination with protein vaccines did not change astrogliosis and microgliosis in brains of T5x mice. The levels of GFAP, P2RY12 and CD45 proteins in the soluble fraction of the brain extracts were analyzed by Western blotting and quantitatively determined by densitometric analysis with normalization against β-actin. The relative protein level in the brains of vaccinated mice is presented as a percentage of the protein level in the brains of control mice. Error bars represent average ± SEM. Statistically significant differences were examined using one-way ANOVA (n = 12 for Control group and n=11 for all vaccinated groups).

Figure S7

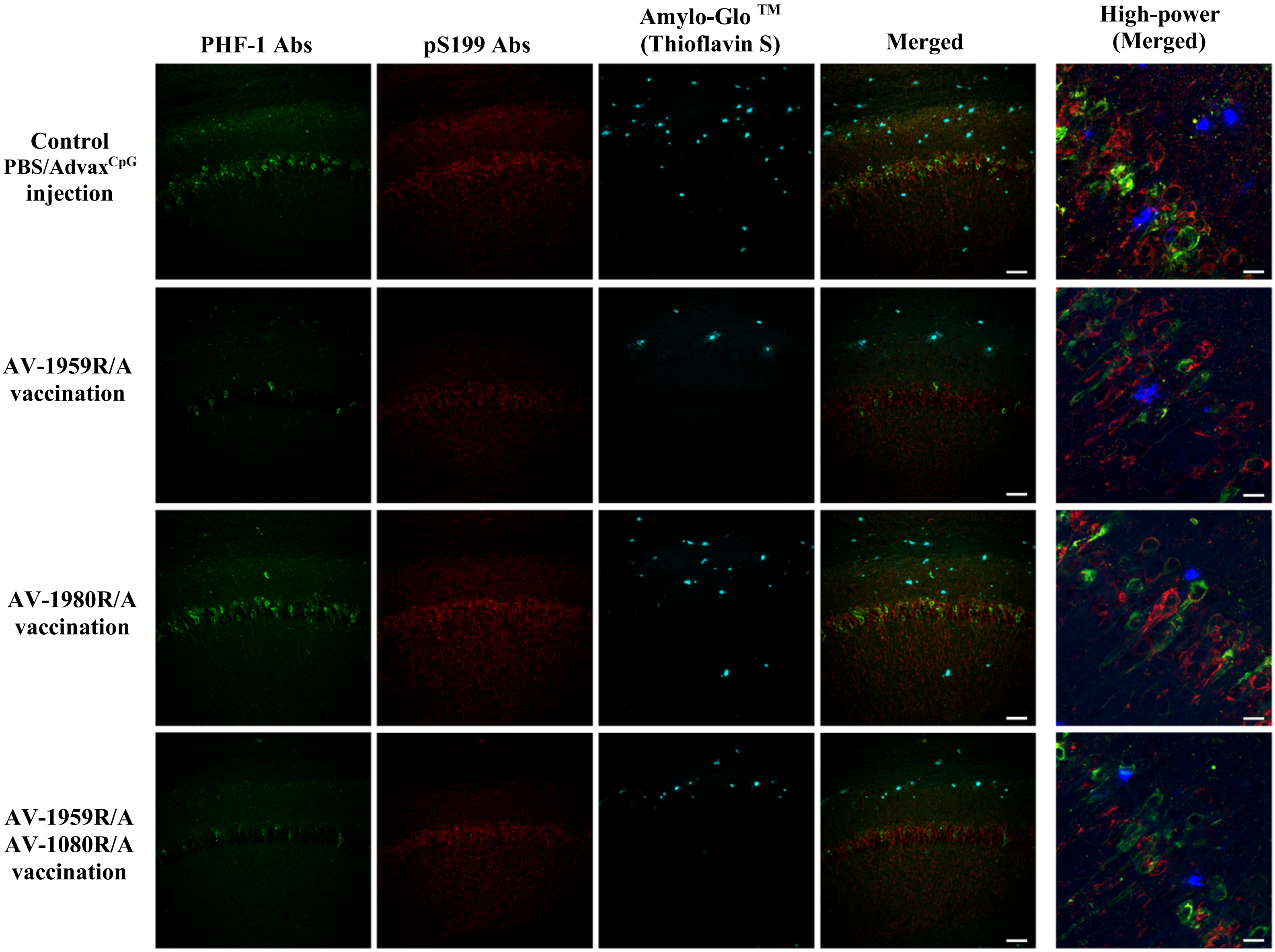

Figure S7. Reduced β-amyloid and tau pathology in T5x mice following vaccination with different proteins. Representative pictures of brain CA1 region immunostained for Amylo-Glo^{TM} (ThS, anti-Aβ) and pS199 and PHF-1 (anti-tau) antibodies. Scale: 60µm (lowpwr) and 15µm (highpwr).
